# Quality of Life Among Patients with Nasal Obstruction—Does Etiology Matter?

**DOI:** 10.3390/jcm15041320

**Published:** 2026-02-07

**Authors:** Lev Chvatinski, Lirit Levi, Amir Levi, Amir Oved, Noam Koch, Aiman El Mograbi, Nimrod Amitai, Itzhak Braverman, Ethan Soudry

**Affiliations:** 1 Department of Otolaryngology Head and Neck Surgery, Hillel Yaffe Medical Center, Ha-Shalom St, Hadera 3820302, Israel; lev.chvatinski@gmail.com (L.C.); braverman1959@gmail.com (I.B.); 2 Rappaport Faculty of Medicine, Technion–Israel Institute of Technology, Efron Street 1, Bat Galim, Haifa 3109601, Israel; 3 Department of Otolaryngology Head and Neck Surgery, Rabin Medical Center-Beilinson Campus, 39 Jabotinsky St., Petah Tikva 4941492, Israel; liritcoh@gmail.com (L.L.); noamcoh@gmail.com (N.K.); aimanelmograbi@gmail.com (A.E.M.); nimrod.amitai@gmail.com (N.A.); 4 Gray Faculty of Medical & Health Sciences, Tel Aviv University, Ramat Aviv, Tel Aviv 6997801, Israel; 5 Sagol School of Neuroscience, Tel Aviv University, Ramat Aviv, Tel Aviv 6997801, Israel; amirlev8@mail.tau.ac.il; 6 Department of Pediatrics, Meir Medical Center, 59 Tchernichovsky St., Kfar Saba 4428164, Israel; amiroved12@gmail.com

**Keywords:** nasal obstruction, allergic rhinitis, deviated nasal septum, inferior turbinates hypertrophy, quality of life

## Abstract

**Objectives:** Nasal obstruction is a common presenting symptom in otolaryngology practice. Frequent etiologies include allergic and non-allergic rhinitis, inferior turbinate hypertrophy (HIT), and nasal septal deviation (DNS). This study aimed to evaluate the relationship between major causes of nasal obstruction and their effect on patient-reported quality of life (QoL). **Methods:** We conducted a retrospective analysis of patients presenting with nasal obstruction who completed the 22-item Sino-Nasal Outcome Test (SNOT-22), the Nasal Obstruction Symptom Evaluation (NOSE) scale, and a visual analog scale (VAS). Patients were categorized into three groups based on etiology: rhinitis, anatomical obstruction, or combined pathology. **Results:** The study included 170 patients (62% male), with a mean age of 38.4 years. Mean SNOT-22, NOSE, and VAS scores were 38, 61, and 6.5, respectively, with no statistically significant differences observed among the three etiologic groups. QoL outcomes were also comparable across anatomical subgroups, including isolated DNS, HIT, or combined findings. Among SNOT-22 domains, rhinologic symptoms demonstrated the highest burden. Patients with rhinitis exhibited significantly higher rhinologic and ear/facial symptom scores compared with patients with isolated anatomical obstruction (*p* = 0.04 and *p* = 0.005, respectively). Strong correlations were observed between SNOT-22, NOSE, and VAS scores across the entire cohort. **Conclusions:** Nasal obstruction is associated with substantial impairment in multiple domains of quality of life, independent of the underlying etiology. These findings highlight the broad impact of nasal obstruction on patient well-being. Larger prospective studies are warranted to further assess changes in quality of life following medical and surgical interventions.

## 1. Introduction

Chronic nasal obstruction is one of the most common otolaryngological complaints [1]. The etiology for obstruction can be anatomical (e.g., nasal septal deviation, inferior turbinate hypertrophy, nasal valve collapse, or adenoidal hypertrophy) or inflammatory (e.g., rhinitis or sinusitis). Chronic rhinitis is an inflammatory disease exclusively affecting the nasal mucosa. Common symptoms are nasal blockage, rhinorrhea, itching, and sneezing. Nasal obstruction in patients suffering from rhinitis is caused by mucosal congestion and swelling, particularly of the inferior nasal turbinates. Chronic rhinitis can be allergic (allergic rhinitis) or non-allergic (non-allergic rhinitis) [1,2]. Studies have shown that allergic rhinitis is associated with QoL impairment, impacting work and school performance as well as sleep quality [3,4].

In contrast to chronic sinusitis and allergy, the effect of nasal obstruction on quality of life measures has been mostly studied in association with surgery and often using one QoL score. In addition, no comparison regarding patients’ QoL has been made between those suffering from anatomical nasal obstruction and those with chronic rhinitis.

The purpose of this study was to comprehensively evaluate QoL measures (SNOT-22, NOSE, VAS) in patients with nasal obstruction either from anatomical obstruction (deviation of the nasal septum and/or inferior turbinate hypertrophy), chronic rhinitis, or both, and to evaluate the correlation between these QoL measures.

## 2. Materials and Methods

### 2.1. Study Design

This study was approved by the local Institutional Ethics Committee and conducted in accordance with the STROBE (Strengthening the Reporting of Observational Studies in Epidemiology) guidelines. The study population included patients ≥ 18-year-old visiting a tertiary rhinology outpatient clinic between the years 2014 and 2021. To be included in this study, patients had to fulfill the following criteria: 1. complaints of nasal obstruction due to one of the following etiologies: chronic rhinitis (symptoms of blockage and runny nose), either allergic (with evidence of allergy in skin tests) or non-allergic (negative skin tests) (NAR) with evidence of inferior turbinate hypertrophy; or anatomical nasal obstruction due to nasal septal deviation and/or hypertrophy of inferior turbinates without symptoms or evidence for allergic or non-allergic rhinitis. Patients with chronic rhinitis were then further stratified into those with and without nasal septal deviation; 2. have filled out quality of life questionnaires (SNOT-22, NOSE questionnaire, and VAS) during their clinic visit. In the VAS (visual analog scale) score, patients were asked to describe how severely their nasal problem affects their quality of life.

Excluded were patients with nasal obstruction associated with other etiologies, such as nasal valve obstruction, chronic sinusitis, adenoid hypertrophy, tumors, as well as patients who did not complete the quality of life questionnaires during the clinical visit.

In addition, SNOT-22 questionnaires, which were collected from a group of CRS patients in the outpatient clinics, were used for comparison to the nasal obstruction only study group.

### 2.2. Data Collection

A computerized scan of the medical records of all patients examined in the otorhinolaryngology department was searched using the following International Classification of Diseases, 9th Revision (ICD-9) diagnoses codes: chronic sinusitis, allergic rhinitis, chronic rhinitis, deviated nasal septum, and inferior turbinate hypertrophy. All identified records were then further evaluated to verify the diagnosis and searched for completed QoL questionnaires (SNOT-22, VAS, NOSE) during the first clinic visit. The cause of nasal obstruction was determined based on history and nasal endoscopy, as determined by the treating physician during the clinic visit.

### 2.3. Statistical Analysis

Data are presented as a median [interquartile range (IQR)] or mean ± standard error of the mean (SEM), as appropriate. Differences between multiple groups were analyzed using the Kruskal–Wallis test with correction for multiple comparisons using Tukey’s procedure. Differences in proportions between two or more categorical variables were assessed using the G-test with correction for multiple comparisons. Correlations between parameters were evaluated using Spearman’s rank correlation coefficient and tested with a permutation test. A *p*-value < 0.05 was considered statistically significant. For all figures, statistical significance is denoted as follows: ns, *p* > 0.05; *, *p* < 0.05; **, *p* < 0.01; ***, *p* < 0.001.

To assess whether quality of life (QoL) scores followed a normal distribution with unknown mean and variance, the Jarque–Bera test was applied. For the overall scores of SNOT-22, NOSE, and VAS, the null hypothesis of normality was rejected (SNOT-22: *p* = 0.02; NOSE: *p* = 0.04; VAS: *p* = 0.02), indicating that the distributions of all three outcome measures were non-normal. Accordingly, non-parametric statistical tests were employed for group comparisons, and results are reported as medians. Upon subgroup analysis of patients with anatomical obstruction, rhinitis, or combined disease, distribution patterns varied between groups, with some demonstrating normal distributions and others not. Therefore, a consistent non-parametric analytical approach was applied across all subgroup comparisons. For comparison of SNOT-22 subdomains with different maximal possible scores (30, 15, 25, 35, and 25), each subdomain score was normalized by dividing the observed score by its maximal possible value, resulting in a normalized range between 0 and 1. During the preparation of this manuscript, the authors used ChatGPT 5.2 (OpenAI, San Francisco, CA, USA) to assist with language editing and phrasing. The tool was not used to generate data, analyze results, or draw scientific conclusions.

## 3. Results

A total of 170 patients with nasal obstruction were included in the study. 106 of them were males (62%), and 64 were females (38%). The mean age was 38.4 ± 1.3 (range 18–88 years). Table 1 demonstrates the epidemiologic characteristics of our cohort. Ninety-six patients were diagnosed with anatomical obstruction (56%), 35 (21%) were diagnosed with chronic rhinitis, and 39 (23%) were diagnosed with both chronic rhinitis and nasal septal deviation (“combined” group).

Out of the 96 patients with anatomical obstruction, 67% (*n* = 64) had a combination of deviated nasal septum and inferior turbinate hypertrophy, while 13% had deviated nasal septum only (*n* = 12), and the remaining 21% (*n* = 20) had solely inferior turbinate hypertrophy. Patients with only anatomical obstruction due to inferior turbinates had no obvious etiology for the enlargement of their inferior turbinates. Approximately 50% of the entire cohort (89 patients) used intranasal corticosteroid sprays. The majority of them (88%) were in the chronic rhinitis group.

### 3.1. QoL Scores

To determine whether quality of life differed between patients with anatomical obstruction, rhinitis, or combined disease, we analyzed SNOT-22, NOSE, and VAS scores across all three groups. In the overall cohort, the mean SNOT-22 score was 38.3 ± 2.1 (range, 1–96), the mean NOSE score was 61.2 ± 1.8 (range, 5–100), and the mean VAS score was 6.5 ± 0.17 (range, 0–10). Group-specific QoL scores are presented in Table 2. No statistically significant differences were observed between the groups for any of the QoL measures (SNOT-22, *p* = 0.50; NOSE, *p* = 0.87; VAS, *p* = 0.67). Further subgroup analysis was performed among patients with anatomical obstruction, stratified into deviated nasal septum (DNS), inferior turbinate hypertrophy (HIT), or combined DNS + HIT. QoL scores for these subgroups are presented in Table 3. No significant differences were identified between the subgroups for any of the QoL measures (SNOT-22, *p* = 0.60; NOSE, *p* = 0.87; VAS, *p* = 0.87).

### 3.2. SNOT-22 Subdomains Comparison

**Figure 1:**
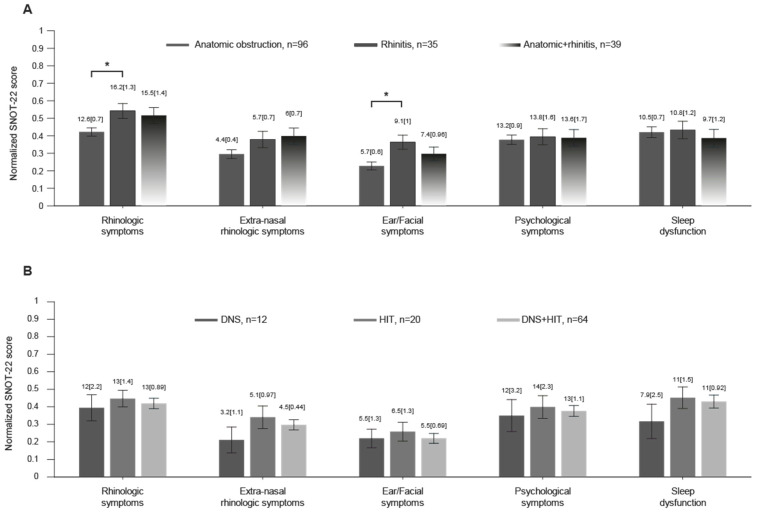
SNOT-22 subdomains histogram—comparison within each subdomain. (**A**) Normalized mean SNOT-22 score of patients with anatomical obstruction, rhinitis, and rhinitis + anatomical obstruction grouped into the five subdomains of SNOT-22. (**B**) Subgroups of patients with anatomical obstruction—DNS, HIT, DNS + HIT. Error bars indicate the standard error of the mean (SEM). Numbers above each bar indicate non-normalized mean ± SEM. *: *p* < 0.05, Kruskal–Wallis test corrected for multiple comparisons.

**Figure 2:**
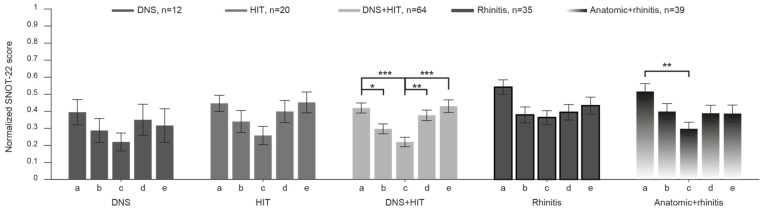
SNOT-22 subdomains histogram—comparison within each subtype. Normalized mean SNOT-22 score of patients with DNS, HIT, DNS + HIT, rhinitis, and rhinitis + anatomical obstruction for each of the five subdomains of SNOT-22. Here, comparison is between subdomain scores for each disease type; a, b, c, d, e indicate the five subdomains of SNOT-22—rhinologic symptoms, extra-nasal rhinologic symptoms, ear/facial symptoms, psychological symptoms, and sleep dysfunction, respectively. */**/***: *p* < 0.05/*p* < 0.01/*p* < 0.001, Kruskal–Wallis test corrected for multiple comparisons.

To further explore potential differences between groups, SNOT-22 subdomain scores were compared across patients with anatomical obstruction, rhinitis, and combined disease (Figure 1). Mean normalized scores were calculated for each of the five SNOT-22 subdomains: rhinologic symptoms, extra-nasal rhinologic symptoms, ear/facial symptoms, psychological symptoms, and sleep dysfunction (Figure 1A). Across all groups, the highest normalized scores were observed in the rhinologic symptoms domain, followed by sleep dysfunction and psychological symptoms (Figure 2). Patients with rhinitis demonstrated significantly higher rhinologic symptom scores and ear/facial symptom scores compared with patients with anatomical obstruction (*p* = 0.04 and *p* = 0.005, respectively). No significant differences were observed between groups for the remaining subdomains. Subdomain analysis among anatomical obstruction subgroups (DNS, HIT, and DNS + HIT) revealed no statistically significant differences across any of the SNOT-22 subdomains.

### 3.3. SNOT-22, NOSE, and VAS Scores for Entire Cohort Are Highly Correlated

**Figure 3:**
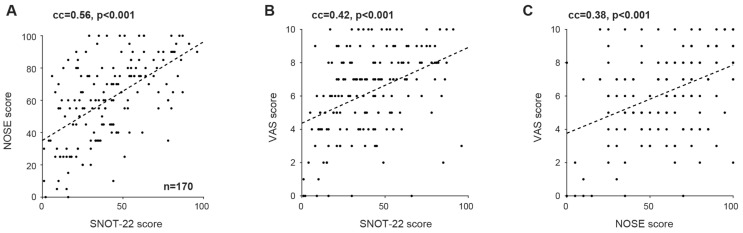
SNOT-22, NOSE, and VAS scores for all patients are highly correlated. (**A**) SNOT-22 scores are correlated with NOSE scores for all 170 patients who undertook both tests. Here and in (**B**,**C**), dashed gray line, linear model fit. cc, rank correlation coefficient. *p* < 0.001, permutation test. (**B**) SNOT-22 scores are correlated with VAS scores. (**C**) NOSE scores are correlated with VAS scores.

Strong correlations were observed between all three QoL instruments. SNOT-22 scores correlated significantly with NOSE scores (Spearman’s rank correlation coefficient ρ = 0.56, *p* < 0.001; permutation test; Figure 3A). Significant correlations were also identified between SNOT-22 and VAS scores (ρ = 0.42, *p* < 0.001; Figure 3B) and between NOSE and VAS scores (ρ = 0.38, *p* < 0.001; Figure 3C).

### 3.4. SNOT-22-CRS vs. Rhinitis and Anatomical Obstruction

**Figure 4:**
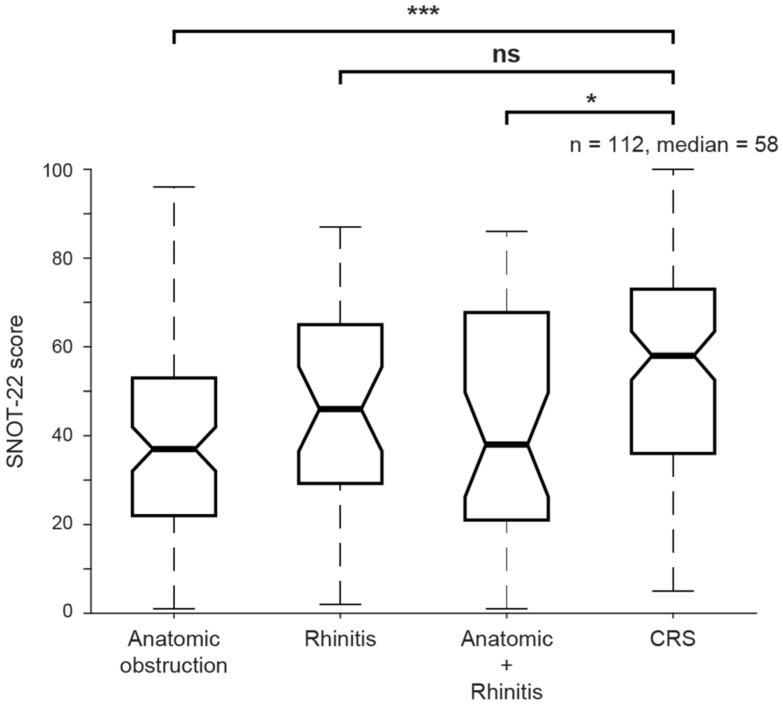
Comparison between SNOT scores in the different nasal obstruction groups and the CRS group. SNOT-22 score of patients with anatomical obstruction, rhinitis, both, or CRS. ns/*/***: *p* > 0.05/*p* < 0.05/*p* < 0.001, Kruskal–Wallis test corrected for multiple comparisons.

To contextualize the SNOT-22 scores observed in the study cohort, scores were compared with those of patients with chronic rhinosinusitis (CRS) who completed the SNOT-22 questionnaire in our outpatient clinic prior to surgical intervention (Figure 4). Patients with CRS demonstrated significantly higher SNOT-22 scores compared with the overall study cohort (median [IQR]: 58 (36–72) vs. 38 (22–56), *p* < 0.001). Further subgroup analysis showed that CRS patients had significantly higher median SNOT-22 scores compared with patients with anatomical obstruction alone (*p* < 0.001) and those with combined rhinitis and anatomical obstruction (*p* = 0.047). No statistically significant difference was observed between patients with rhinitis alone and those with CRS (*p* = 0.30)

## 4. Discussion

In this study, we found that nasal obstruction is associated with a significant impairment in quality of life as reflected in the NOSE, VAS, and SNOT-22 questionnaires. Interestingly, we found no major differences in the scores of these questionnaires between the different patient groups, emphasizing nasal obstruction as a fundamental symptom affecting patients’ quality of life. Nonetheless, these questionnaires seem complementary, specifically SNOT-22, which adds more information on several aspects of sinonasal quality of life as demonstrated by the differences in subdomain scores depending on the etiology of obstruction [5,6].

The impact and severity of nasal obstruction symptomatology as perceived by patients were assessed using the Nasal Obstruction Symptom Evaluation (NOSE) scale described by Stewart et al. in 2004 [7]. On the final measure, results range from 0 to 20. By multiplying the raw score by 5, the test was scaled to a total score of 0 to 100. A score of 0 indicates no nasal blockage, and a score of 100 indicates the worst conceivable nasal obstruction [7]. A systematic review of patients with nasal obstruction who underwent surgical procedures by Rhee and colleagues found that the overall mean NOSE score in these patients before intervention was 65 and that in asymptomatic patients, the average score was 15 [8]. Alanazy et al. reported the preoperative differences in QoL between patients with and without allergic rhinitis and patients who underwent septoplasty with or without turbinates reduction. The mean overall preoperative NOSE questionnaire score was 58.4, with a mean score in the allergy group of 65.4 compared to 54.6 in those without allergy [9]. In the present study, we also found high NOSE scores (mean 61.2) in all patient groups, with a mean score of 65 in the combined group versus 60 in the other groups; however, with no statistically significant differences across all three sub-groups. This is consistent with previous studies looking at septoplasty outcomes in patients with and without allergic rhinitis, showing similar preoperative scores [10,11]. Nonetheless, as the majority of patients with rhinitis received INCS treatment, higher NOSE scores may have been documented in these patients if such treatment had not been given. A recent study evaluated NOSE scores for patients scheduled for nasal septum repair and inferior turbinoplasty and observed a mean score of 72.1 [12], higher than observed in our study, which also included patients not scheduled for surgery. The average VAS scores have been observed to range from 6.33 to 7.48 in patients with nasal obstruction due to nasal septal deviation [13,14]. In this study, looking at other causes of nasal obstruction, we observed similar results (mean 6.5) with no differences between the nasal obstruction subgroups. The SNOT-22 questionnaire was created to assess disease-specific quality of life in patients suffering from chronic sinusitis [15]. Average scores in healthy subjects were observed to be 10.2 in males and 13.2 in females [16]. Hopkins et al. followed 3128 patients with chronic sinusitis with and without polyposis who underwent endoscopic sinus surgery and found that the mean preoperative score was 40.9 [17]. In the Israeli population, average SNOT-22 scores in healthy population were observed to be 13.15, and 50.44 in chronic sinusitis patients [18]. Assessment of QoL using the SNOT-22 questionnaire in non-CRS patients was described by Hoehle et al. in patients with allergic rhinitis [3]. In their study, they observed that the mean scores in these patients were as high as 36.9 in the SNOT-22 questionnaire and 46.9 in the NOSE questionnaire. Similar to our study, a correlation between SNOT-22 and NOSE scores was found in patients suffering from allergic rhinitis. Another study from the same group examined QoL indices in two patient groups: one group with allergic rhinitis treated with intranasal corticosteroid sprays (INCS) and a second group with allergic rhinitis without treatment. QoL scores were tested using questionnaires: SNOT-22, VAS, and Rhinitis Control Assessment Test (RCAT). The mean SNOT-22 was found to be similar, with the group that used INCS being 41.8 compared to the group that did not use the spray, which was 35.6 [19]. In our study, we observed that the overall mean SNOT-22 score of patients suffering from nasal obstruction was 38.3, which was significantly higher than the average healthy scores in our country. This highlights the significant impact of nasal obstruction on the quality of life of patients. However, when compared to a cohort of CRS patients in our clinic (mean score of 58), this score was significantly lower. Interestingly, although SNOT-22 scores were not significantly different between the different patient groups, the rhinitis group had the highest mean score (mean 46), which was not statistically significant compared to CRS patients. We were then further interested in identifying whether there were specific domains on the SNOT-22 questionnaire that were more dominant in patients’ QoL. We observed, as may be expected, that the rhinological domain received the worst scores. Interestingly, the psychological domain received the next worst score, followed by the sleep dysfunction domain, underscoring the substantial impact of nasal obstruction on mood and sleep quality. In contrast, Hohle, looking at allergic rhinitis patients, observed the worst scores for the ear/facial symptoms [3]. Speth et al., evaluating the SNOT-22 questionnaire to examine the various symptoms of allergic rhinitis, found that there were no statistically significant differences between the extra-nasal symptoms and the rhinological symptoms [19]. Nonetheless, to the best of our knowledge, the present study is the first to evaluate QoL measures in patients with nasal obstruction and to compare them between the three most common etiologies separately and combined.

### Study Limitations

As this study was conducted in tertiary rhinology outpatient clinics, approximately half of the patients were already on intranasal corticosteroid (INCS) treatment, particularly chronic rhinitis patients, and as such, it is possible that the impairment of their QoL was even worse than reflected in our results, and further emphasizes the impact of nasal obstruction on quality of life. Also, as the study is retrospective, the patient groups were not equal in size and may not represent the entire patient population, as inclusion was limited to patients with available completed questionnaires. Moreover, seasonal variability and duration of symptoms were not uniformly controlled across groups, which may have influenced patient-reported outcomes. In addition, the unequal group sizes and the small number of patients with isolated septal deviation limited the strength of subgroup-level conclusions. Furthermore, this study reflects quality of life assessment at presentation and did not evaluate post-treatment outcomes. Finally, the exclusive use of patient-reported outcome measures without objective nasal airflow assessment may introduce subjectivity. Nonetheless, to the best of our knowledge, the present study is the first to evaluate QoL measures in patients with nasal obstruction and to compare them between the three most common etiologies separately and combined.

## 5. Conclusions

In this study, we found significant impairment in the QoL of patients with nasal obstruction affecting both rhinological and non-rhinological (mainly psychological and sleep) aspects of quality of life, regardless of obstruction etiology. Furthermore, SNOT-22, NOSE, and VAS scores were found to be highly correlated in these patients. Further studies are needed on larger groups of patients, including analysis of expected improvement following medical and surgical treatment.

## Figures and Tables

**Table 1:** Clinical and demographic characteristics of the study participants.

Characteristics		Clinical Diagnosis			Total	*p*-Value
		Anatomical Obstruction	Rhinitis	Rhinitis + Nasal Septal Deviation		
Study Participants		96 patients	35 patients	39 patients	170 patients	
	**Gender**, *n* [%]					
	Males	65 (61%)	20 (19%)	21 (20%)	106	0.42
	Females	31 (49%)	15 (23%)	18 (28%)	64	
	**Age** (years) mean ± SEM	37.2 ± 1.7	40.9 ± 3.1	39.1 ± 2.8	38.4 ± 1.3	0.84

Note: SEM = standard error of the mean.

**Table 2:** SNOT-22, NOSE, and VAS scores of patients with anatomical obstruction, rhinitis, and a combination of anatomical obstruction and rhinitis.

SNOT-22, NOSE and VAS Mean and Median Scores		Anatomic Obstruction (*n* = 96)	Rhinitis (*n* = 35)	Rhinitis + Septal Deviation (*n* = 39)	*p*-Value
SNOT-22	mean ± SEM	38.6 (2.13)	45.8 (4)	43 (4.2)	0.21
	Median [IQR]	37 (22, 53)	46 (23, 66)	38 (21, 69)	0.5
NOSE	mean ± SEM	61.2 (2.4)	59 (4.3)	58.7 (4.2)	0.83
	Median [IQR]	60 (45, 80)	60 (45, 80)	65 (35, 80)	0.87
VAS	mean ± SEM	6.5 (0.2)	5.7 (0.5)	5.9 (0.4)	0.22
	Median [IQR]	7 (5, 8)	6 (3, 8)	6 (4, 8)	0.67

SEM = standard error of mean. IQR = interquartile range.

**Table 3:** SNOT-22, NOSE, and VAS scores of patients with anatomical obstruction subgroups: deviated nasal septum (DNS), hypertrophy of inferior turbinate (HIT), or both.

SNOT-22, NOSE and VAS-Mean and Median Scores		DNS (*n* = 12)	HIT (*n* = 20)	HIT + DNS (*n* = 64)	*p*-Value
SNOT-22	mean ± SEM	34 (7.1)	41.2 (4.4)	38.3 (2.6)	0.6
	Median [IQR]	23.5 (16, 43)	40 (28, 47)	37.5 (22, 53)	0.6
NOSE	mean ± SEM	60.4 (9.1)	57.7 (5.5)	62.4 (2.8)	0.75
	Median [IQR]	60 (25, 85)	57.5 (35, 70)	60 (50, 80)	0.87
VAS	mean ± SEM	6.3 (0.9)	6.9 (0.3)	6.4 (0.3)	0.64
	Median [IQR]	6.5 (4, 9)	7 (6, 8)	7 (4, 8)	0.87

SEM = standard error of mean. IQR = interquartile range.

## Data Availability

The data presented in this study are available on request from the corresponding author due to privacy and ethical restrictions.
